# Reply to the letter from Dr. Pirjo Pärnänen *et al*

**DOI:** 10.14814/phy2.14743

**Published:** 2021-02-01

**Authors:** Mukulika Bose, Bhaskar Mitra, Pinku Mukherjee

**Affiliations:** ^1^ University of North Carolina at Charlotte Charlotte NC USA; ^2^ Idaho National Laboratory Idaho Falls ID USA

## Abstract

Nutraceuticals could be used to increase immunity against viral infections and therefore more research should be done on them to better manage COVID‐19 and future pandemics

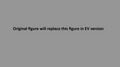

We appreciate the letter provided by Dr. Pärnänen et al., in response to our article (Bose et al., 2021), and thank the Editor‐in‐Chief for allowing us to reply. The authors have put forward an excellent hypothesis that polyphenols could provide immunity against corona virus infectious disease‐19 (COVID‐19).

Polyphenols are bioactive substances that have been shown to be beneficial to oral health, lung functions, asthma, chronic obstructive pulmonary disease (COPD), and intestinal health and have triggered more investigation. They modulate immune responses in different diseases as reviewed previously (Ding et al., 2018). Polyphenols derived from plums may target the Akt/mTOR pathway and microRNA 143, both of which have been identified as potential factors in colon cancer tumorigenesis (Banerjee et al., 2016). As mentioned in the letter, a key component of the COVID‐19 inflammatory pathophysiology is the upregulation of MAPK pathway, therefore providing a link to assess the efficacy of polyphenols in preventing severe disease outcome in COVID‐19 patients. Polyphenols in red wine can significantly increase the level of interleukin‐21 (IL‐21) and decrease the release of pro‐inflammatory cytokines IL‐1β and IL‐6 (Magrone et al., 2017). They enhanced intestinal mucosal immunity by increasing the populations of intraepithelial T cells and mucosal eosinophils during helminth infection in pigs (Williams et al., 2017). The immune index of IgA in the guts of rats that were fed a high‐fat diet was shown to have been significantly increased by curcumin (Okazaki et al., 2010). Therefore, the concept of using polyphenols from lingonberries as a nutraceutical tool to prevent COVID‐19 disease is worth exploration.

Whether polyphenols provide protection against COVID‐19 or any other infection should be confirmed by human intervention trials. Before these trials are designed, the effect of polyphenols on the oral cavity should be studied and a safety assessment of the applied dose should be performed (Lolayekar & Shanbhag, 2012). Investigation on the suitable time, dose, and means of polyphenols is needed to optimize their functions in human subjects. Many epidemiological studies have shown that the consumption of these compounds can reduce the incidence of chronic diseases but the actual results from the intervention experiments have not matched the expected results. The reasons for this discrepancy are not fully understood. However, there are many factors that have made research on polyphenols extremely difficult, including structural diversity, lack of standardized methods to analyze the content, and variation of content in the same food product. These create difficulty to estimate the average daily intake of polyphenols with precision. Therefore, a more comprehensive survey of the occurrence of different types of polyphenols in food must be performed using well‐standardized methods. Most studies that provide evidence of the potential of polyphenols to prevent diseases are in vitro or animal experiments, which use much higher doses than those consumed by humans through diet. Moreover, to maintain a high concentration of polyphenols in plasma, one needs to repeatedly ingest the product over time. However, the bioavailability of polyphenols in the plasma of colon in the form of unknown metabolites is longer. These must be produced either in our tissues or by the colonic microflora. Hence, the role of the microflora in the bioavailability of polyphenols needs to be assessed. The diversity in the microbial composition of the colon could explain the interindividual variations in bioavailability of polyphenols. Polyphenols affect immunological responses and can not only regulate the host immune system but also target the pathogen. There are many factors influencing susceptibility to infection, disease progression, and response to therapy for both infectious and chronic diseases. These factors include nutrition (Bennett et al., 2015), microbiome composition (Bose & Mukherjee, 2019), and host mucin glycosylation (Bose & Mukherjee, 2020). Polyphenols play an important role in the maintenance of microbial community and these microbes promote the oxidation and degradation of polyphenols. Therefore, polyphenols may modulate the immune responses of the host by altering the microbiota. In summary, regular consumption of foods or beverages abundant in polyphenols may help maintain overall health effectively. With regard to prevention of infections, studies on humans are needed to confirm the reports provided by many experimental studies. Better knowledge of the structure, dose, and bioavailability of dietary polyphenols will be critical to properly evaluate their role in the prevention of infection in future. If these challenges are overcome, polyphenols could offer a cost‐effective public health intervention in preventing infections. Therefore, we believe that more research on the efficacy of polyphenols as nutraceutical tools should be encouraged to abate the effect of future pandemics.

## CONFLICT OF INTEREST

The authors declare no conflict of interest.
